# Case report: Osteo-oto-hepato-enteric syndrome caused by UNC45A deficiency

**DOI:** 10.3389/fgene.2022.1079481

**Published:** 2023-01-09

**Authors:** Ruixue Wang, Yizhong Wang, Ronghua Yu, Wuhen Xu, Ting Zhang, Yongmei Xiao

**Affiliations:** ^1^ Department of Gastroenterology, Hepatology, and Nutrition, Shanghai Children’s Hospital, School of Medicine, Shanghai Jiao Tong University, Shanghai, China; ^2^ Gut Microbiota and Metabolic Research Center, Immunity and Critical Care Medicine, School of Medicine, Institute of Pediatric Infection, Shanghai Jiao Tong University, Shanghai, China; ^3^ Molecular Diagnostic Laboratory, School of Medicine, Shanghai Children’s Hospital, Shanghai Jiao Tong University, Shanghai, China

**Keywords:** O2HE, cholestasis, compound heterozygote variants, UNC45A, case report

## Abstract

**Background:** Recently, UNC45 myosin chaperone A (UNC45A) deficiency was identified as a cause of osteo-oto-hepato-enteric syndrome (O2HE) characterized by congenital diarrhea, neonatal cholestasis, deafness, and bone fragility. To date, only a few O2HE cases have been reported in the literature.

**Case presentation:** Here, we present a child from China diagnosed with O2HE with novel compound heterozygous variants in *UNC45A*. The patient suffered with neonatal jaundice, cholestasis, and intractable diarrhea after birth. Laboratory tests revealed highly elevated levels of total serum bilirubin (TB), direct bilirubin (DB), and total bile acid (TBA). The patient was managed with ursodeoxycholic acid (UDCA)-based treatments, and the clinical symptoms and abnormal liver functions were significantly relieved. The patient’s hearing was normal, and no sign of bone fragility was observed. Exome sequencing (ES) identified novel compound heterozygote variants c.292C>T (p.Arg98Trp)/c.2534-2545del (p.Leu845-Met848del) in *UNC45A*, which were inherited from her mother and father, respectively. Both variants are predicted to be deleterious by *in silico* predictors.

**Conclusion:** We present an O2HE child from China with novel compound heterozygous variants in *UNC45A*. Our patient’s clinical manifestations were less severe than those of the previous reported cases, which expands the clinical spectrum of O2HE.

## Background

Neonatal cholestasis is defined as the impairment of bile formation or flow that is characterized by intrahepatic cholestasis, jaundice, and conjugated hyperbilirubinemia in newborns and young infants (Feldman and Sokol, 2020). The incidence of neonatal cholestasis is approximately 1:2500 live births (Fawaz et al., 2017). As a multifactorial disease, more than 100 hepatobiliary and/or metabolic disorders have been clarified that are responsible for the occurrence of neonatal cholestasis (Feldman and Sokol, 2020). The leading cause of neonatal cholestasis is biliary atresia (BA), which accounts for 25%–40% of the total cases, and followed by monogenic disorders (∼25%) (Feldman and Sokol, 2020). Defects in different genes related to the regulation of bile acid synthesis, secretion, transport, and metabolism have been linked to neonatal cholestasis in last decades, including pathogenic mutations in bile acid synthesis and conjugating enzyme-coding genes (*AKR1D1*, *CYP7A1*, *CYP7B1*, *SLC27A5*, etc.), genes coding for canalicular transporters (*ATP8B1*, *ABCB11*, *ABCB4*, etc.), and nuclear hormone receptor gene, *NR1H4* (Lam et al., 2010; Paulusma et al., 2010; Srivastava, 2014; Gordo-Gilart et al., 2015; Feldman and Sokol, 2020). In addition, deficiencies in genes of cytoskeleton and tight junctions were identified to be associated with neonatal cholestasis, such as mutations in *MYO5B* and *TJP2* (Sambrotta et al., 2014; Qiu et al., 2017; Ge et al., 2019). However, known gene defects cannot account for all hereditary cholestasis cases, and many patients remain genetically undiagnosed.

Recently, osteo-oto-hepato-enteric syndrome (O2HE) presenting with congenital diarrhea, neonatal cholestasis, deafness, and bone fragility attributed to loss-of-function mutations in UNC45 myosin chaperone A (*UNC45A*) was described (Esteve et al., 2018; Duclaux-Loras et al., 2022). Here, we report a male infant from China presented with neonatal cholestasis and intractable diarrhea caused by novel compound heterozygous variants of *UNC45A*.

## Case presentation

A three-month-old boy was admitted to our hospital because of a history of jaundice and intractable diarrhea after birth. The boy was born full-term by cesarean section with a birth weight of 2,250 g. He is the second child of a healthy non-consanguineous couple of Chinese Han ethnicity. The family history was unremarkable. His five-year old brother was healthy. Mild jaundice of the skin and the sclera were observed after birth, and diarrhea was characterized by loose, yellow–green, non-bloody stools occurring 3–5 times per day. He was diagnosed with neonatal jaundice and was discharged from the neonatology department after improvement in the serum bilirubin level. However, jaundice and diarrhea persisted, and he was admitted to the local hospital again when he was 3 months old. Laboratory tests showed highly elevated levels of total serum bilirubin (TB 137.8 μmol/L, reference range: 3.4–17.1 μmol/L), direct bilirubin (DB 107.8 μmol/L, reference range: 0–6.8 μmol/L), and total bile acid (TBA 217.3 μmol/L, reference range: 0–10 μmol/L), and then he was transferred to our hospital for further evaluation.

On admission, the patient’s body temperature was 36.8°C, heart rate was 137 per min, respiratory rate was 27 per min, and blood pressure was 88/57 mmHg. He had a normal weight of 4.8 kg and a height of 58 cm. The skin of the face, trunk, and limbs and the sclera were mildly yellow. The heart and lungs were normal, the abdomen was soft, the liver was 4 cm below the ribs, and the spleen was not touched under the ribs. The muscle strength and tension of the limbs and the neurological examination were unremarkable. The binaural auditory brainstem response (ABR) wave V threshold was 25 dBHL. The liver biochemical profile revealed elevated levels of alanine aminotransferase (ALT 391 U/L, reference range: 5–40 U/L), aspartate aminotransferase (AST 565 U/L, reference range: 8–40 U/L), gamma-glutamyltransferase (GGT 81 U/L, reference range: 7–32 U/L), TB 136.7 μmol/L (reference range: 3.4–17.1 μmol/L), DB 104.4 μmol/L (reference range: 0–6.8 μmol/L), and TBA 195 μmol/L (reference range: 0–10 μmol/L) (Table 1). The detection of liver-damaging pathogens was negative, including Epstein–Barr virus (EBV), cytomegalovirus (CMV), herpes simplex virus (HSV), and hepatitis A, B, and C viruses. Abdominal ultrasound indicated hepatomegaly with diffuse changes. Hepatobiliary scintigraphy with single-photon emission computed tomography (SPECT) revealed impaired hepatic uptake and clearance function and delayed biliary excretion. Magnetic resonance imaging (MRI) showed that the gallbladder was filled, and the common bile duct was less visible. BA was excluded by laparoscopic common bile duct exploration. The diagnosis of intrahepatic cholestasis was made, and the patient was treated with symptomatic therapies of oral ursodeoxycholic acid (UDCA, 50 mg/day), fat-soluble vitamins (vitamin AD, 1800 U/day; vitamin E, 25 mg/day), intravenous compound glycyrrhizin (15 mg/day), and ademetionine 1, 4-butanedisulfonate (250 mg/day).

**TABLE 1:** Liver biochemical profile of the patient.

Biochemical index	Reference	3 months	4 months	6 months	9 months	12 months
TB (μmol/L)	3.4–17.1	136.75	78.56	12.49	3.9	5.9
DB (μmol/L)	0–6.8	104.38	42.3	3.0	1.4	2.15
ALT (U/L)	5–40	391	156	68	45	35
AST (U/L)	8–40	565	176	74	49	54
TBA (μmol/L)	0–10	195	138	11	16.22	12.62
GGT (U/L)	7–32	81	110	42	23	18

TB, total serum bilirubin; DB, direct bilirubin; ALT, alanine aminotransferase; AST, aspartate aminotransferase; GGT, gamma-glutamyltransferase; TBA, total serum bile acid.

**FIGURE 1:**
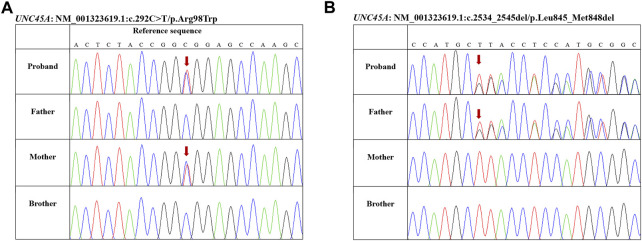
Genotyping of the family members by Sanger sequencing. **(A)** Proband and his mother carrying variant c.292C>T (p.Arg98Trp). **(B)** Proband and his father carrying variant c.2534-2545del (p.Leu845-Met848del).

To further investigate the etiology of intrahepatic cholestasis, exome sequencing (ES) using genomic DNA extracted from peripheral blood was performed on a HiSeqX10 (Illumina, United States) platform. Novel compound heterozygote variants c.292C>T (p.Arg98Trp)/c.2534-2545del (p.Leu845-Met848del) in *UNC45A* were identified and were further confirmed by Sanger sequencing (Figure 1). No other potentially pathogenic variants were observed in known genes related to cholestasis. Genotyping of the unaffected family members by Sanger sequencing showed that his mother carries the c.292C>T (p.Arg98Trp) variant, his father carries the c.2534-2545del (p.Leu845-Met848del) variant, and both variants are not detected in his brother (Figure 1). Variant c.292C>T (p.Arg98Trp) is found four times (4/282,568) in heterozygotes, and c.2534-2545del (p.Leu845-Met848del) is reported only once (1/250,666) in the heterozygote state in the gnomAD database. *In silico* tools predicted that the c.292C>T (p.Arg98Trp) missense variant is deleterious (PROVEAN score, -6.989; PolyPhen-2 score, 1.000). The c.2534-2545del (p.Leu845-Met848del) variant leads to a deletion of four amino acids in the C-terminal UCS domain, and it is predicted to be deleterious with a PROVEAN score of -16.547.

The patient was discharged with alleviated jaundice and significantly improved liver function indexes after 1 month of symptomatic treatments. Maintenance treatments with oral UDCA (50 mg/day), compound glycyrrhizin (12.5 mg/day), and fat-soluble vitamins (vitamin AD, 1800 U/day; vitamin E, 25 mg/day) were recommended to be continued further after discharge from the hospital. During the recent 9-month follow-up period, he had complete clinical and biochemical recovery except for slightly elevated levels of AST and TBA (Table 1). At this writing, he is 1 year old and remains stable with a normal growth (weight, 8,750 g; height, 74 cm) after drug withdrawal for 3 months. He can walk with the help of an instrument, and no sign of bone fragility was observed. His language development is normal, and repeated hearing testing was unremarkable.

## Discussion

As a clinical feature common to numbers of hepatobiliary and metabolic diseases, neonatal cholestasis is a condition that requires prompt diagnosis for achieving optimal outcomes. Genetic testing is widely used to define the hereditary etiology of neonatal cholestasis cases without anatomic obstruction of the biliary system. Recently, loss-of-function mutations in *UNC45A* was reported to be related with a syndrome named O2HE associating cholestasis, diarrhea, impaired hearing, and bone fragility (Esteve et al., 2018; Duclaux-Loras et al., 2022). To the best of our knowledge, we are the first to report an O2HE case from China with novel compound heterozygous variants in *UNC45A*. The main clinical manifestation of our patient was neonatal cholestasis and persistent diarrhea. ES identified novel compound heterozygote variants c.292C>T (p.Arg98Trp)/c.2534-2545del (p.Leu845-Met848del) in *UNC45A*, which were inherited from his mother and father, respectively. Both variants were defined as damaging/likely pathogenic according to the guidelines of the American College of Medical Genetics (ACMG) (Kalia et al., 2017).

To date, a total of 10 O2HE patients from eight families with *UNC45A* loss-of-function mutations were described in the literature (Esteve et al., 2018; Duclaux-Loras et al., 2022). We briefly summarized the characteristics of the reported O2HE patients with *UNC45A* mutations in Table 2 *.* The most common clinical symptoms of the reported O2HE patients were severe diarrhea (9/10), followed by neonatal cholestasis (8/10), and bone fracture and deafness were presented in six and four patients, respectively (Table 2). Parenteral nutrition and enteral nutrition were required to treat severe diarrhea. In contrast, our patient only suffered with mild diarrhea (3–5 times/day) and recovered along with cholestasis relief at the age of 6 months. In addition to oral UDCA and fat-soluble vitamins, the patient was treated with intravenous compound glycyrrhizin and ademetionine 1, 4-butanedisulfonate. Glycyrrhizin is an active ingredient extracted from licorice, a widely used herbal medicine in traditional Chinese medicine with hepatoprotective and detoxifying properties (Li et al., 2019). The active metabolic product of glycyrrhizin, glycyrrhetinic acid, could relieve cholestasis by regulating bile acid transporters and is anti-inflammatory (Li et al., 2019; Yan et al., 2021). Ademetionine 1, 4-butanedisulfonate injection is a preparation with S-adenosyl-L-methionine (SAMe) as the main active ingredient. SAMe is an important methyl donor that participates in multiple cellular methyltransferase reactions present for an article type that does not allow ones reactions (Lieber and Packer, 2002). It has been shown that SAMe supplementation restores hepatic glutathione (GSH) deposits and attenuates liver injury (Lieber and Packer, 2002; Mato and Lu, 2007). Both compound glycyrrhizin and ademetionine 1, 4-butanedisulfonate injections are recommended for the treatment of intrahepatic cholestasis in China (National Center for Clinical Research of InfectiousD, 2022). Since some O2HE patients’ hearing loss was diagnosed in the late age, further hearing testing is necessary to determine the possible hearing impairment of the patient. At the time of this writing, no sign of bone fragility was observed. Although the patient has remained stable for 3 months with normal growth after discontinuation of drug treatment, he should be monitored closely to be aware of symptom recurrence and late-onset manifestations. For genetic variants in *UNC45A*, a total of 15 variants were observed, including four homozygous variants and seven compound heterozygote variants, and no genotype and phenotype correlations were identified (Table 2).

**TABLE 2:** Summary of clinical and genetic features of mutations in the *UNC45A* leading to O2HE.

	Variant and consequence	Origin	Gender	Onset age	Age at writing	Diarrhea	Cholestasis	Bone fragility	Deafness
P1^[10]^	c.784C>T:p.Arg262*	European descent	Girl	4 days	5 years	Yes, need TPN	No	No	Yes
	c.1268T>A:p.Val423Asp								
P2^[10]^	c.2581C>T:p.Gln861*	Tunisia	Girl	15 days	23 years	No	Yes, resolved at 2.5 years	23 times bone fractures	Yes, diagnosed at 5 years
	c.2633C>T:p.Ser878Leu								
	c.2734T>G:p.Cys912GIy								
P3^[10]^	c.2581C>T:p.Gln861*	Tunisia	Girl	7 days	18 years	Yes, need TPN	Yes, resolved at 3 years	Yes	Yes, diagnosed in the teens
	c.2633C>T:p.Ser878Leu								
	c.2734T>G:p.Cys912GIy								
P4^[10]^	c.247C>T:p.Arg83Trp	Turkish	Girl	15 days	5 years	Yes, need TPN	Yes, resolved at 3 years	Yes, two times bone fractures	No
	c.983G>T:p.Gly328Val								
P5^[11]^	c.710T>C:p.Leu237Pro (homozygous)	Turkish	Girl	3 weeks	6 years	Yes, hypovolemic shock	Yes	Yes, two times bone fractures	No
P6^[11]^	c.721C>T:p.Arg241*	United Kingdom	Girl	1 week	2 years	Yes, need TPN	No	No	No
	c.2182G>A:p.Glu728Lys								
P7^[11]^	c.1452delinsGCA:p.Asp484Glufs*17	French	Boy	1 week	22 years	Yes, need EEN, intermittent until 10 years	Yes	Yes, two times bone fracture	Yes
	c.2512G>C:p.Ala838Pro								
P8^[11]^	c.689C>G:p.Thr230Arg (homozygous)	French (West Indies)	Girl	4 days	10.5 years	Yes, need TPN and small bowl transplantation	No	No	No

P9^[11]^	c.710T>C:p.Leu237Pro (homozygous)	Turkish	Girl	1 day	Died at day 93	Yes, need TPN	Yes	ND	No
P10^[11]^	c.710T>C:P.Leu237Pro (homozygous)	Turkish	Girl	5 days	3 months	Yes, need TPN	Yes	Yes, one time bone fracture	ND
P11^a^	c.292C>T:p.Arg98Trp	China	Boy	1 day	1 year	Yes, resolved at 6 months	Yes, resolved at 6 months	No	Yes
	c.2534-2545del:p.Leu845-Met848del								

^a^ Case reported in this study. UNC45A, UNC45 myosin chaperone A; EEN, exclusive enteral nutrition; ND, not determined; TPN, total parenteral nutrition; O2HE, osteo-oto-hepato-enteric syndrome.

UNC45 is a member of the evolutionary conserved UNC45/Cro1/She4p (UCS) protein family. As an important myosin cochaperone, UNC45 participates in the regulation of a variety of myosin-driven processes, such as cytokinesis, endocytosis, and muscle organization (Lee et al., 2014). There are two related UNC45 proteins (UNC45A and UNC45B) in vertebrates with a sequence identity of 55%, and UNC45A is a ubiquitously expressed protein in different organs, while UNC45B is only expressed in muscle cells (Lee et al., 2014). UNC45 contains three recognizable domains: a C-terminal UCS domain that binds to myosin II motor, a central domain with unknown function, and an N-terminal tetratricopeptide repeat (TPR) domain that interacts with heat shock protein 90 (Hsp90) (Venolia et al., 1999).

Functional studies revealed that loss-of-function mutations in *UNC45A* attenuate or abolish UNC45A activity, leading to gut development and functional defects (Esteve et al., 2018). Lechuga et al. (2022) showed that defects in UNC45A disrupted epithelial barrier integrity, impaired the assembly of epithelial adherence and tight junctions, and attenuated cell migration by disorganizing actomyosin bundles at epithelial junctions and the migrating cell edge. It has been shown that the loss expression of *UNC45A* or replacement of the endogenous *UNC45A* gene with the *UNC45A* gene carrying the loss-of-function mutation associated with O2HE led to a significantly reduced expression of its cochaperone, myosin VB, in intestinal and liver epithelial cells (Li et al., 2022). As mentioned previously, O2HE patients displayed heterogeneous clinical features, and no obvious genotype and phonotype correlations were observed. Similarly, the heterogeneity presentation has been described in MYO5B-associated microvillus inclusion disease (MVID) patients (Perry et al., 2014). The loss of myosin VB expression is most likely responsible for severe diarrhea in MVID. In accordance with other reported cytoskeleton and tight junction gene defects associated with intrahepatic cholestasis (*MYO5B* and *TJP2*) (Sambrotta et al., 2014; Qiu et al., 2017; Ge et al., 2019), cholestasis presented in O2HE patients may be caused by *UNC45A* mutation-mediated epithelial barrier integrity disruption. Furthermore, mutated UNC45A may affect other myosin proteins involved in precise arrays of mechanosensitive microvilli-like stereocilia crowning the auditory hair cells (e.g., myosin IIIa and MYO15A) that lead to hearing loss (Lelli et al., 2016; Li et al., 2022). UNC45A is required for proper folding of MYO15A. MYO15A is necessary for elongation and maintenance of inner ear hair cell stereocilia (Bird et al., 2014). Stereocilia are required for mechanotransduction of sound. Variants of MYO15A are associated with human deafness DFNB3 (Wang et al., 1998). Thus, UNC45A deficiency-related O2HE is considered a new variant of MVID (Duclaux-Loras et al., 2022).

## Conclusion

In summary, we are the first to report a Chinese male O2HE child with novel compound heterozygous variants in *UNC45A*. The clinical manifestations of our patient were less severe than those of the previous reported cases, which expands the clinical spectrum of O2HE. Future studies are needed to further investigate the molecular mechanisms of UNC45A deficiency.

## Data Availability

The datasets for this article are not publicly available due to concerns regarding participant/patient anonymity. Requests to access the datasets should be directed to the corresponding author.

## Abbreviations

ABCB, adenosine triphosphate-binding cassette subfamily B; AKR1D1, steroid A-ring 5 beta-reductase; ALT, alanine aminotransferase; AST, aspartate aminotransferase; ATP8B1, ATPase class I type 8B member 1; BA, biliary atresia; CYP7, cholesterol 7alpha-hydroxylase; DB, direct bilirubin; EBV, Epstein–Barr virus; ES, exome sequencing; GGT, gamma-glutamyltransferase; MVID, microvillus inclusion disease; MYO5B, motor myosin VB; NR1H4, nuclear receptor subfamily 1, group H, member 4; O2HE, osteo-oto-hepato-enteric syndrome; SLC27A5, solute carrier family 27 member 5; TB, total serum bilirubin; TBA, total bile acid; TJP2, tight junction protein 2; TPR, tetratricopeptide repeat; UDCA, ursodeoxycholic acid; UNC45A, UNC45 myosin chaperone A.
